# Hepatic Disorders, from a Brief Treatise on Therapeutics

**Published:** 1885-05-20

**Authors:** J. Milner Fothergill


### HEPATIC DISORDERS, FROM A BRIEF TREATISE ON THERAPEUTICS.

BY J. MILNER FOTHERGILL, M.D., M.R.C.P.

Of agents which stimulate the liver, and bring away bile-laden stools, are, conspicuously, mercurials, ipecacuanha and sulphate of soda. Euonymin is, also, a fairly potent hepatic stimulant. Now, you all know that the liver is the furnace in which waste and spare albuminoids are oxidized into urea and uric acid. The bile acids—glycocholic and tauro-cholic—are also nitrogenized bodies. The functions of the liver and kidneys are closely linked together; and in those derangements where the urine has a thick sediment and the bowels are disordered, the old-fashioned doctor, who shook his head and oracularly uttered, "Liver!" was not such a fool as it has recently been the rule to regard him. There is nitrogenized waste in the blood; so do two things: First, cut down the amount of albuminoids eaten or drank, in order to reduce the demand upon the liver; then sweep away the waste from the blood by a pill at bedtime:

℞ Pulv. pip. nig.......................grs. ij
Pil. cal. col. co.......................grs. iij, and in the morning:

℞ Sodæ pot. tart.......................ʒ j

Sodæ sulphatis.......................℥ ss

Tinct. zingiberis.......................ʒ ss

Inf. gentian.......................℥ j, with an equal quantity of boiling water, so as to make the draught as hot as can be comfortably borne. Let this be done twice or thrice a week till the tongue is clean. When that is done, give the

℞ Sodæ sulphat.......................ʒ i

Sodæ pot tart.......................℥ ss

Tinct. nuc. vom.......................gtt. vj

Inf. cascarillæ.......................℥ j,

Ter in die.

before meals, and the pill twice a week.

If there be general asthenia, do not proceed to give iron until the tongue is thoroughly clean, the water clear, and the appetite good: and then commence with two or three drops of the dialysed iron once a day after food. Here you wish to give the iron as a hæmatic only: if you give it in tonic doses, it will upset the assimilative processes, and disagree with the patient to a moral certainty.

In other cases, where there is only slight constipation, with deposits in the urine, especially after meals, give the old-fashioned dinner pill:

℞ Pulv. ipecacuanha........................grs. j
Pulv. capsici........................grs. ss
Ext. cinchonæ........................grs. iij
Pil. al. et myrrh........................grs. j, every day after dinner. It will be found very efficacious. If this dinner pill does not act sufficiently, give the morning laxative twice or thrice a week, so long as the bowels require it.

[Of these laxatives, or liver stimulants, we know of nothing better than elixir frangulaxine in dessertspoonful doses before breakfast or dinner.]

Then, as to the union of laxatives with tonics, it is well often to combine these two agents. In convalescence, tonics never act genially if there be not at the same time regular and sufficient action of the bowels; so, add sulphate of magnesia or sulphate of soda to the tonic,

℞ Mag. sulphat........................℈ j

Vel sodæ sulphat........................ʒ j

Quin. sulph........................grs. j

Ac. phosp. dil........................m. xv

Inf. gentian........................℥ j,

Ter in die, before meals, and ten minims of dialysed iron after dinner daily will usually give good results; or,

℞ Mag. sulphat........................ʒ j

Tinct. fer mur........................m. x

Liq. strychniæ........................m. iv

Inf. quass........................℥ j,

Ter in die, forms a less expensive form of tonic, of much utility.

As other tonics in gouty subjects, or those who are rheumatic, or recovering from acute gout or rheumatism, it may be desirable to give,

℞ Mag. sulph........................℈ j

Pot. bicarb........................℈ ss

Fer. am. cit........................grs. v

Inf. quass........................℥ j,

Ter in die, before meals, with a draught of water. (The importance of dilution has been pointed out in a previous chapter.) If necessary, give the Pil. Cal. Col. Co. once or twice a week at bedtime.

But in this use of laxatives, with occasional mercurials, avoid the pitfall of letting the patient eat with unlicensed abandon. To sweep away nitrogenized waste, and so relieve the assimilative process from the accumulation of debris, is to improve the appetite; just as a fire burns up when the ashes, which interfere with oxidation, are poked out. But if the dietary be not, at the same time, regulated, and the albuminoids cut down, the condition is a very undesirable one, and the double stimulation of the assimilative processes by too liberal supplies of albuminoid food on the one hand, and the exhibition of hepatic stimulant-laxatives on the other, will in time land the patient in a very unhappy state, from which it will not be easy to rescue him. This therapeutic plan can cut both ways, according to the dietary adopted.

To stimulate the liver, it is at times well to place a large hot poultice over the right side: this is a measure the astute student will do well to bear in mind.

When the tongue is very foul, it may be well on the second morning of the treatment to give

℞ Calomel ........................................ grs. iv
Pulv. jalapæ ........................................ grs. xv, and so open the bowels freely. This usually brings away copious bile-laden evacuations, after which the tongue cleans under the night pill and the day medicine (previously recommended). Sometimes these morning powders have to be repeated at intervals of three or four days.

When in ascites, or dropsy, or in the constipation of meningitis, it becomes necessary to resort to cathartics,

℞ Elaterin........................................ grs. 1-20

Pulv. jalapæ........................................ ʒ ss, or,

℞ Pulv. gambogiæ........................................ grs. iij

Pulv. pip. nigr........................................ ℈ ss

Potass. bitartrat........................................ ʒ ij, may be given every second morning, along with the appropriate measures, for meningitis and for dropsy. In all these cases free catharsis gives great relief.

Now, in conclusion, let me tell the student to strive to see what are the indications for treatment; what is most prominent, pain, sleeplessness, fever, collapse, hæmorrhage, dyspnœa; what, in this case, calls most imperiously for attention. He is taught too exclusively, at present, to look at disease from a dead-house point of view. To make a diagnosis which would be corroborated in the dead-house is the great matter! Yes, so it is at a medical school; but in practice for yourself, remember that a living, grateful patient, who has got well under your care, is worth far, far more to you than any amount of accurate diagnosis—which, so far as other persons and their opinions are concerned, is as voiceless to further your interests as the tombstones in the churchyard which mark your failures.—*Leonard's Med. Monthly.*

